# Preferences and priorities to manage clinical uncertainty for older people with frailty and multimorbidity: a discrete choice experiment and stakeholder consultations

**DOI:** 10.1186/s12877-021-02480-8

**Published:** 2021-10-14

**Authors:** India Tunnard, Deokhee Yi, Clare Ellis-Smith, Marsha Dawkins, Irene J. Higginson, Catherine J. Evans

**Affiliations:** 1 King’s College London, Cicely Saunders Institute of Palliative Care, Policy and Rehabilitation, Bessemer Road, London, SE5 9PJ England; 2 Sussex Community NHS Foundation Trust, Brighton General Hospital, Elm Grove, Brighton, BN2 3EW England

**Keywords:** Intermediate care facilities, Uncertainty, Frailty, Aged, 80 and over, Aged, Caregivers, Comorbidity, Health communication, Palliative care, Observational study

## Abstract

**Background:**

Clinical uncertainty is inherent for people with frailty and multimorbidity. Depleted physiological reserves increase vulnerability to a decline in health and adverse outcomes from a stressor event. Evidence-based tools can improve care processes and outcomes, but little is known about priorities to deliver care for older people with frailty and multimorbidity. This study aimed to explore the preferences and priorities for patients, family carers and healthcare practitioners to enhance care processes of comprehensive assessment, communication and continuity of care in managing clinical uncertainty using evidence-based tools.

**Methods:**

A parallel mixed method observational study in four inpatient intermediate care units (community hospitals) for patients in transition between hospital and home. We used a discrete choice experiment (DCE) to examine patient and family preferences and priorities on the attributes of enhanced services; and stakeholder consultations with practitioners to discuss and generate recommendations on using tools to augment care processes. Data analysis used logit modelling in the DCE, and framework analysis for consultation data.

**Results:**

Thirty-three patients participated in the DCE (mean age 84 years, SD 7.76). Patients preferred a service where family were contacted on admission and discharge (*β* 0.36, 95% CI 0.10 to 0.61), care received closer to home (*β* − 0.04, 95% CI − 0.06 to − 0.02) and the GP is fully informed about care (*β* 0.29, 95% CI 0.05–0.52).

Four stakeholder consultations (*n* = 48 participants) generated 20 recommendations centred around three main themes: tailoring care processes to manage multiple care needs for an ageing population with frailty and multimorbidity; the importance of ongoing communication with patient and family; and clear and concise evidence-based tools to enhance communication between clinical teams and continuity of care on discharge.

**Conclusion:**

Family engagement is vital to manage clinical uncertainty. Both patients and practitioners prioritise engaging the family to support person-centred care and continuity of care within and across care settings. Patients wished to maximise family involvement by enabling their support with a preference for care close to home. Evidence-based tools used across disciplines and services can provide a shared succinct language to facilitate communication and continuity of care at points of transition in care settings.

**Supplementary Information:**

The online version contains supplementary material available at 10.1186/s12877-021-02480-8.

## Introduction

As the population is living longer, they are often doing so with frailty and multimorbidity [1–3]. Family members (unpaid caregivers close to the patient, including friends) are important to the care of this population and particularly in enabling patients to achieve their wishes for care, such as, care and support to remain at home. Both frailty and multimorbidity inherently increase vulnerability to an uncertain illness trajectory. This trajectory is marked by seemingly minor stressor events, such as an infection, causing a disproportionate decline in health and function [3]. These marked declines are characterised with uncertainty around outcomes of treatment, prognosis and nearness for end-of-life. If clinical uncertainty is poorly managed it can lead to negative outcomes for the patient and their family [4, 5] and impact upon care delivery [6].

Clinical uncertainty is conceptualised in four main types: 1) Complexity; 2) Unpredictability; 3) Ambiguity; and 4) Lack of information [7–9]. These four types of uncertainty hinder a patient’s ability to understand their condition, in turn increasing uncertainty. Etkind et al. [10] built on this conceptualisation from the patient perspective, developing a typology detailing that determines a patient’s response to uncertainty. This includes: levels of engagement (how involved with their condition and care the patient wishes to be); temporal focus (period in time the patient is focused on); and information preferences (level of information desired by patient). Further, Goodman et al. [11] theorised uncertainty in older people as something that practitioners should ‘hold’, rather than seek to resolve to better manage care in care homes. A systematic review [12] by the authors constructed a conceptual framework building on these conceptual understandings to identify core care processes to address clinical uncertainty and better manage care for adults with frailty and multimorbidity. The framework comprised three core care processes within uncertainty: 1) Comprehensive assessment; 2) Communication of uncertainty; and 3) Continuity of care. The review examined evidence of tools for clinical care, using processes to identify patient priorities and needs, such as the Comprehensive Geriatric Assessment, and tools for continuity of care, such as discharge pathways and advance care planning, to enhance integrated care. Using tools to enhance care processes was considered a way to optimise care and patient outcomes. However, communication of clinical uncertainty had the least evidence and was challenging to do well.

The use of evidence-based tools to improve care processes and outcomes is well established. Evidence is convincing on the value of using standardised tools in the care of older adults to improve assessment, such as the Comprehensive Geriatric Assessment for older adults in hospital [13], using validated outcome measures to identify unrecognised symptoms [14, 15], and advance care planning to identify and communicate preferences and priorities at the end of life [16]. However, much of the research evidence concerns acute care interventions to manage clinical uncertainty. Tools to support the management of clinical uncertainty have been developed mainly in the acute hospital, such as the AmberCare Bundle on managing care towards the end of life [17], and the PACE tool on communication with the family in intensive care [18]. Evidence is limited in intermediate care settings that are key in managing clinical uncertainty for older people with frailty and multimorbidity [19]. These facilities care for mainly adults aged 80 years and over, with multimorbidity, where uncertainty is inherent, and following an unplanned admission. Over one in four die within a year of their index admission [20]. These settings are key for older people to support recovery and rehabilitation, and plan for and anticipate nearness of end of life [21, 22]. Intermediate care settings include care of the elderly wards, nursing homes and community hospitals. We focus on community hospitals as the care settings managing older people’s care at points of transition between hospital and home (or care home). Community hospitals provide mainly post-acute care for older people often following an unplanned acute hospital admission [23, 24]. This is a point of greatest need to support recovery, prevent further hospitalisations and plan for nearness of end of life.

This study aims to explore patient and family priorities and preferences for enhanced care processes to manage clinical uncertainty for older people in community hospitals. And to explore practitioners’ priorities and preferences for managing clinical uncertainty using evidence-based tools.

### Objectives

- To identify the patient preferences and priorities to manage clinical uncertainty and the relative importance of attributes to enhance care processes
- To test the feasibility of the DCE of patient and family carer recruitment, and acceptability of the design
- To explore practitioner priorities to manage clinical uncertainty using standardised tools
- To integrate findings to inform a conceptual model on managing clinical uncertainty for older people with frailty and multimorbidity

## Methods

### Study design

We used a mixed methods parallel observational study design incorporating a discrete choice experiment (DCE) with cognitive interviews, and stakeholder consultations. The study was underpinned by our conceptual framework of clinical uncertainty [12] developed from a theoretical understanding on uncertainty in illness [7–11], and systematic reviews on models of care [22] and tools for managing clinical uncertainty for older people towards the end of life [12].

The study was approved by London - Camberwell St Giles Research Ethics Committee, London (REC reference 18/LO/1343). The reporting of this study conforms to the STROBE checklist [25] (Supplementary file 1).

### Setting

Four community hospitals providing intermediate care to older people located in urban localities in South England between October and December 2018. Hospitals had one or two wards with an average of 23 beds per ward. The area has a larger than UK average percentage of over 65 s (25% vs 18% UK, 2016 estimates), 88.9% of the population were white British and the level of deprivation was low [26, 27].

### Discrete choice experiment

A DCE was used to explore patient and family’s preferences and priorities for a service to manage clinical uncertainty. A DCE is a quantitative attribute-based method of measuring benefit that assumes participants prioritise a service from its attributes rather than the service itself [28]. The attributes and levels were informed by underpinning theory of clinical uncertainty, preliminary findings from the stakeholder consultation, the project steering committee and public involvement members. Attributes included: *Timing of communication*; *Topics to discuss*; *Timing and mode of communication with family*; *Communication with GP*; and *Distance to community hospital*. Each attribute had between 4 and 6 levels. Choice sets defining two hypothetical services were formed from a combination of levels within each of the four attributes (Supplementary file 2. Further methods). Participants were given eight choice sets to complete. The DCE questionnaire also asked participants about the care they have received, to rate the difficulty of the DCE (1 = *extremely easy* to 5 = *extremely difficult*) and provide their demographic information, such as ethnicity, place of residence and marital status.

We aimed to recruit 33 patients and 10–13 family members. The sample size estimate is based on minimum 30 participants required for analysis with 10% attrition. Eligibility criteria included: aged 65 years and over, able to give informed consent and an inpatient in a participating hospital; or a family carer (including friends) of a participating patient, aged 18 years and over and able to provide informed consent. Community hospital notes were screened and information on reason for admission and diagnoses recorded. Eligible patients were identified and approached by the clinical team/research nurse. Family members were identified by eligible patients. Consent was taken by the research nurse who administered the DCE questionnaire in an interview and assessed patients’ level of functional ability using the Australian-modfied Karnofsky Performance Status (AKPS) scale [29]. We piloted the DCE with five participants using cognitive interviewing with participants talking through their decision making and understanding of the DCE. The pilot explored the clarity of the DCE attributes and levels, comprehension and engagement, and the number of choice sets. The cognitive interviews were digitally recorded and transcribed. The narrative qualitative data allowed for further exploration of the preferences indicated by participants in the quantitative data.

### Stakeholder consultations

Four consultations were held in four community hospitals, with a planned sample of 5–12 participants per consultation to stimulate discussion and allow for all to contribute [30]. Practitioners were purposively sampled from each hospital to ensure representation of disciplines and grades including staff located in the hospital, organisational clinical leads and external services, such as social workers and service commissioners. Those unable to attend consultations were offered an individual interview. An invitation letter/email and research briefing document were sent to eligible practitioners. All participants gave informed consent.

Stakeholder consultations used a modified nominal group technique. The nominal group technique [31] brought together experts to generate recommendations and agree priorities on tool characteristics and strategies to enable use. This technique was modified to include focus groups using a topic guide prior to generation of recommendations. Consultations were facilitated by experienced clinical academics in nursing and occupational therapy (CJE, CES). Facilitators opened the consultation by exploring the context of delivering care in a community hospital. This served to orientate participants on the relevant population and inform understanding of the context and interpretation of findings. The nominal group concerned two main questions:

- **Question 1:** What are the key characteristics of tools for clinical practice to enhance care processes of comprehensive assessment, communication with families and continuity of care between hospital and home (or care home)?
- **Question 2:** How can we enable practitioners to use tools in clinical practice to enhance care processes of comprehensive assessment, communication with families and continuity of care between hospital and home (or care home)?

Each question was discussed separately and exhaustively. Participants then wrote and ranked individual recommendations before providing their top recommendations to the group. Each group discussed the top recommendations given and agreed their top 3. Consultations were recorded and transcribed verbatim.

### Data analysis and integration

#### Discrete choice experiment

Participant characteristics were described and data were analysed using a conditioinal logit model. Only complete choice sets were included in analysis, missing data were excluded. Sign and relative magnitude of coefficients estimated in the regression analysis were used to understand preference. Number of miles willing travel to the hospital providing a service with specific attributes or levels was estimated from the marginal rate of substitution, using the ratio of two coeffiicients, one being on *Distance to community hospital*.

Cognitive interview transcripts were analysed using thematic analysis [32] with data management supported by Excel software. Analysis involved exploration of preferences and acceptability of DCE design. DCE quantitative data (from questionnaire) and qualitative data (from cognitive interviews) were integrated at the point of analysis and data categorised into domains of uncertainty [33].

#### Stakeholder consultations

Transcripts were analysed using Framework Analysis [34] (supplementary file 3) in Nvivo 12 (QSR International, Warrington, UK). Codes were informed by the underpinning theoretical understanding of clinical uncertainty [7–12], familiarisation with data and research team discussions. Coding was completed by a single researcher (IT), with 20% of coding independently checked by a second researcher (CJE) and discussed to ensure consistency. Coding was further discussed and agreed with the wider research team. Participants’ individual recommendations and rankings were entered into an Excel spreadsheet. Flip chart notes containing top recommendations were typed, noting agreed top 3. These recommendations were deduplicated and combined by a single researcher (IT) and reviewed by a second researcher (CE). Final set of recommendations were agreed by ≥1 groups or given by ≥3 participants across ≥2 groups. Findings were triangulated at point of analysis where convergence was sought [33]. Findings from the discrete choice experiment and stakeholder consultations were triangulated at the point of interpretation [33].

## Results

### Discrete choice experiment

**Fig. 1:**
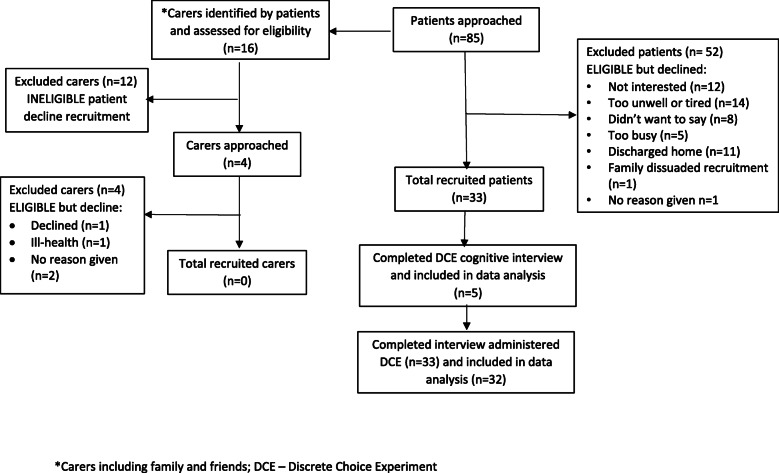
Flow diagram of patient and carer recruitment for the DCE

Thirty-three patients were recruited from 85 approached. Main reasons for patient decline were too unwell (*n* = 14) and discharged (*n* = 11) (Fig. 1). Participants and non-participants were similar in characteristics (Supplementary file 4). Only 16 of the 85 patients approached could identify a family carer (18.8%). No carers were recruited with most ineligible as the patient declined recruitment (*n* = 12), and the four eligible carers declined. Five patients participated in a cognitive interview and were representative of the wider sample. All patients participated in the interview administered DCE. Participants were admitted from an acute hospital (91%), with mean age 84 years (SD 7.76) and AKPS performance status median 50%, indicating they required considerable assistance and frequent medical care (range 50–60) (Table 1). The main reason for admission was a fall (64%). Most participants (94%) had more than one recorded diagnosis, main conditions were circulatory diseases (24.6%) and musculoskeletal diseases (23.1%) (Supplementary file 4). Participants (*n* = 32) indicated their priorities during the admission were for staff to understand ‘what matters to me’ (97%), to have sufficient information (94%) and involvement in decisions (94%) about their care and treatment. Only 56% reported being asked by staff ‘what matters to you’. Priority outcomes post-discharge were to live as independently as possible (100%) at home (97%), and for the GP to be fully informed about individual priorities and preferences, health conditions and management (94%) (Supplementary file 5).

**Table 1:** Patient participant characteristics for the DCE

*Patient characteristics (N = 33)*	
Age, Mean (SD)	84 years (SD 7.76)
Australian-modified Karnofsky Performance Status scale, Median % (range: min - max)	50% (50–60)
Gender, n (%)	
Female	25 (75.8)
Male	8 (24.2)
Ethnicity, n (%)	
White British	31 (93.9)
White – other	2 (6.1)
Marital status, n (%)	
Widowed	15 (45.5)
Married/Civil partnership	8 (24.2)
Single	7 (21.2)
Divorced/separated	3 (9.1)
Living situation, n (%)	
Alone	22 (66.7)
With spouse/partner	8 (24.2)
With other e.g. friend	3 (9)
Place of residence in last 3 months, n (%)	
Own home	28 (84.8)
Home of relative or friend	1 (3)
Other: hospital	4 (12.1)
Admit to community hospital from, n (%)	
Acute hospital	31 (91)
Home	1 (3)
Other community hospital	1 (3)

### Feasibility and acceptability of DCE

On average it took 51 min to complete the DCE. Participants were able to complete eight (*n* = 25; 75.8%) or six (*n* = 5; 15.1%) choice sets. However, two participants completed two or less choice sets and one participant could not complete any. Overall, participants found making choices moderately difficult (mean 3.29; SD 1.13). Participants completed the DCE with a research nurse to support comprehension by reading aloud the choice sets and talking through the hypothetical nature of the DCE. Participants expressed hesitancy when a scenario was inconsistent with their experiences such as *‘Straight away I will not go for one because it’s only 5 miles and there isn’t a community one within 5 miles as far as I can see’* [ID B02023] (Table 2, DCE 0.5–0.8). The cognitive interviews illustrated participants’ consideration and difficulty at times in choosing between services, but this also demonstrated engagement with the survey (Table 2, DCE.07). Despite these minor difficulties in comprehension, participants considered the survey as comprehensive, covering all aspects of community hospital care (Table 2, DCE.08).

**Table 2:** Illustrative quotes from discrete choice experiment cognitive interviews and stakeholder consultation focus groups

Theme	Code	Quote and participant ID code
*Discrete Choice Experiment cognitive interviews*		
**Comprehensive assessment**	DCE.01	*‘… .although I mean it should be carried on throughout I think, whatever, I don’t think that should be an option, it should be discussed because there have been times that I’ve come in here and on my assessment day I’ve been too out of it to talk about anything’ B02022*
**Continuous communication**	DCE.02	*‘Yeah and, you know, the family think, I wouldn’t want them out of it completely, if they wanted to come in and discuss things I’d like it to be an option yeah but not feel that they were pressurised into it …’ B02022*
	DCE.03	*‘yes, the distance was a big influence on me, erm, and also the communication with the family, I didn’t like it when there was no communication’* B03021
**Continuity of care**	DCE.04	*‘There’s this problem here again with GPs, erm, I had to fill in a form today to say whom my GP was, well I’ve got a named GP which everybody has but I very rarely see her because she only works one day a week now’ B02022*
**Acceptability of the DCE**	DCE.05	‘*Well you see, I’m not ill am I? I’m only just injured’ B03023* *‘Yes, yeah’ Interviewer* *‘So then I don’t really feel that level of care’ B03023*
	DCE.06	*‘Straight away I will not go for one because it’s only 5 miles and there isn’t a community one within 5 miles as far as I can see’* B02023
	DCE.07	*‘So once again I’m drawn to service B but there’s no phoning when admitted, I don’t know, it’s interesting that I find that so important isn’t it?’* B02021
	DCE.08	*‘It’s a brilliant idea, it’s wonderful [indecipherable at 12:50] staff to do it, it’s nothing to do with me but it’s wonderful’* B02023
*Stakeholder consultations focus groups*		
**Comprehensive Assessment: ‘Managing an increasingly older population and uncertain outcomes’**		
*Change in patient care needs and practice with an increasingly older population*	SC.01	‘*I think patients that previously would have come here are being discharged directly home from the acute hospitals and being supported at home because of the services that are now in place in the community and whether we’re getting patients that would have typically remained in the acute hospitals but actually because they’re not needing any acute interventions, we’re having to manage them here and they are, …*., *medically complex and often quite on a knife edge where it doesn’t take very much at all to tip them over where you’ll have to put a lot of medical interventions to keep them here and not transfer them back into the acute’* A02003 Advanced Nurse Practitioner
*Person-centred focus on gathering key information*	SC.02	*‘It’s about how you come to a decision about when it’s time to move that person on whether it’s that they, if they progress better by going home sooner and having support in the community for that individual or whether they need to stay here and have another 6 weeks of walking practice, you know, it’s what is going to help that person get to their optimum and I don’t know if it’s a tool thing, I don’t think it’s a simple tool, I think it’s a combination of effectively working and yeah, agreeing a plan and working out something together it’s not one person doing their tick box kind of thing’* Participant in group 03
	SC.03	‘*It’s very frequently, patients referred for rehabilitation and it’s very, very clear when they arrive that they’re not for rehabilitation and actually there hasn’t been those conversations so they come here for rehabilitation [and end-of-life care] because no one’s had time to have those conversations previously and it happens an awful lot doesn’t it?*’ Participant in group 02
**Communication: ‘Continuous communication to manage care’**		
*Continuous communication with the family to support and inform care planning*	SC.04	*‘a lot of the conversations that we have to have, probably for every day, we’re pretty much having a meeting with a family to say, this is where they’re at, they’re not as good as they were previously, so, you know, we’re recommending A, B and C to, you know, you know, they may not be appropriate to return home or, they’re going to need this and this to manage at home, and they can be really challenging conversations to have’* A04001 Physiotherapist
*Supporting patient’s psychological adaption to change in ‘what I can do’*	SC.05	*‘that would be the time to be honest because people are quite often coming over here for intensive rehab … when actually the poor patient you see sitting in front of you, it’s quite obvious that that’s not going to happen so erm, and spending the time at that initial assessment you find what direction you’re actually going in then, it’s the time to be honest and to gather a lot of information’* A02016 Ward sister
	SC.06	*‘Yeah there’s* [not] *a magic front door at home that cures all ills when they get there’* Participant in Group 01
**Continuity of Care: ‘Using evidence-based tools to enhance continuity of care’**		
*Managing care at points of transition in care settings*	SC.07	*‘one of my ideas was very similar to the therapy point of view where they have a sort of health passport document where it could be with the patient or it could be as a part of their notes but where is vital information that we struggle to find’* A01015 Staff nurse
*Tension between nationally standardised tools and flexibility in use*	SC.08	*‘making the information movable between care settings so the information is fluent and the same’* A01008 Older people mental health liaison
	SC.09	*‘it has to be fit for purpose and the staff need to want to fill it in and it needs to be accurate’* A02016 Ward sister
	SC.10	*‘how do you make sure that there’s that kind of local ownership and things grow locally, erm, but it doesn’t become so there’s a post code lottery’* A02019 Commissioner

### Patient priorities for care and relative importance of care processes and components

**Fig. 2:**
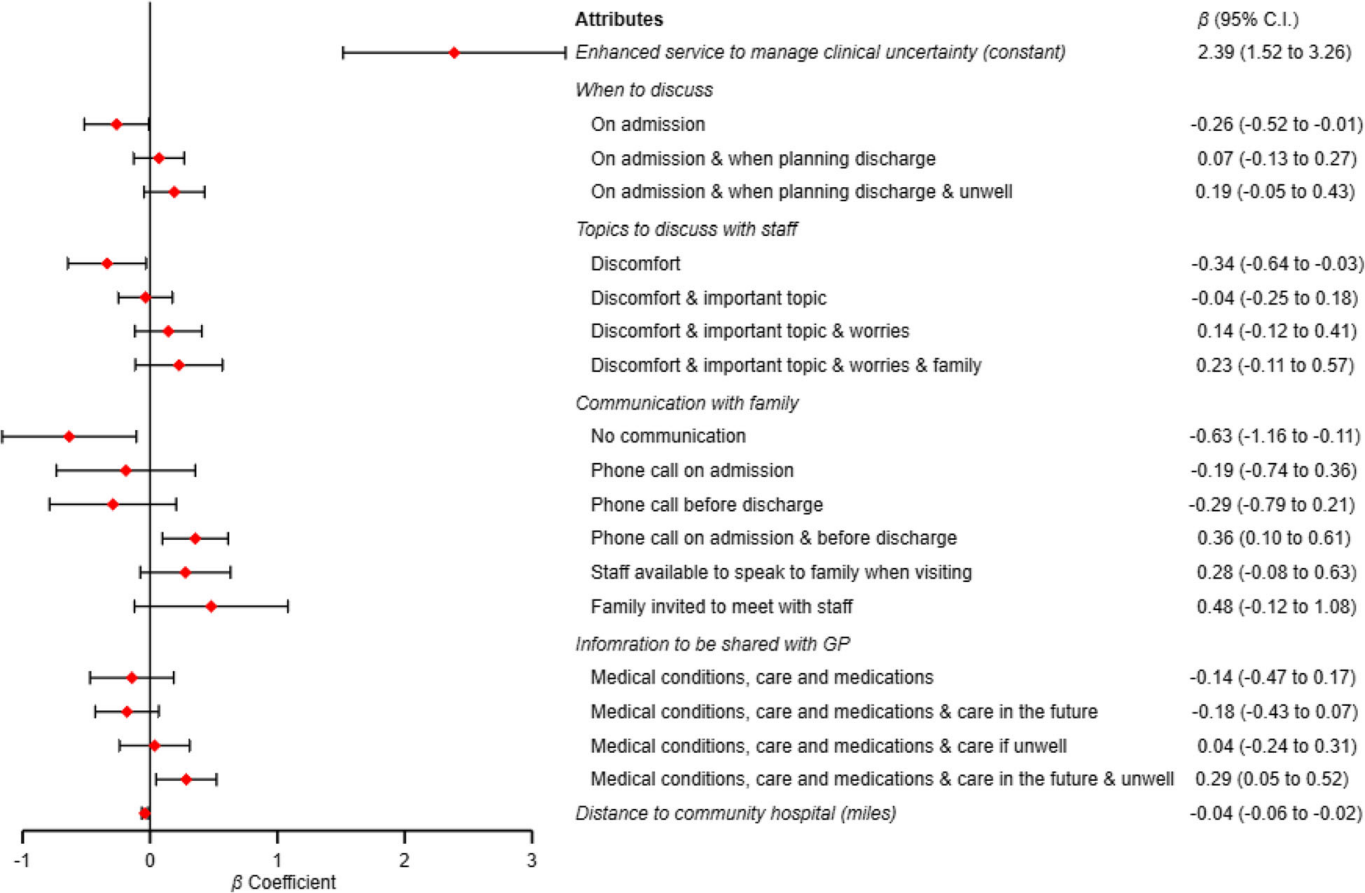
Preference weights from Discrete Choice Experiment (*n* = 32, 699 observations)*. *Missing data *n* = 1 participant could not complete DCE

Data analysis included 32 cases with one missing with participant unable to complete the DCE. The DCE choice sets provided 699 observations for analysis, enabling statistical interpretation of the data. Overall, participants preferred an enhanced service to manage clinical uncertainty (*β* 2.39, 95% CI 1.52–3.26). This large and statistically significant constant term indicates strong acceptability for the services we proposed. See Fig. 2 for all preference weights. Findings on patient preferences and priorities are considered within the domains of uncertainty of ‘*comprehensive assessment’*, ‘*communication’* and ‘*continuity of care’*.

#### Comprehensive assessment

Preferences on the timing of a comprehensive assessment were equivocal. Participants did not prefer the service discussing concerns and preferences only upon admission (*β* − 0.26, 95% CI -0.52 - -0.01). However, they did not show a preference for a specific time proposed. Qualitative data indicated that participants preferred continuous communication of concerns and preferences during their stay, not at fixed points (Table 2, DCE.01). Comprehensive assessment discussions of patient discomfort alone were not preferred (*β* − 0.34, 95% CI -0.64 - -0.03). Participants wanted to discuss as many topics as possible beyond discomfort, including shared decision-making and gaining further information. Changes in the magnitude of coefficients on levels in this attribute showed a gradual increase, reflecting the additive nature of the levels to encompass the breadth of health domains and individual priorities. However, descriptive survey questions indicated that 22% of participants did not prioritise discussions about preferences for future care and 50% did not desire this as an outcome of their admission to the community hospital (Supplementary file 5, Tables S6 and S7).

#### Communication with family

Communication with family about care and treatment was a priority. No communication was not preferable (*β* − 0.63, 95% CI -1.16- -0.11) (Table 2, DCE.02). Patients preferred a service that phoned the family on admission and before discharge and were willing to travel for nine more miles to get this service. Levels ‘Staff available to speak to family when visiting’ and ‘Family invited to meet with staff’ were also preferred, but the confidence intervals were too wide to be assured (Fig. 2). Patients indicated a preference to stay closer to home to allow family to visit (*β* − 0.04 95% CI -0.06 - -0.02). The cognitive interviews echoed this, patients wanted their family involved in decisions about their care, but in ways that minimised burden, such as a shorter distance to travel to visit (Table 2, DCE.03).

#### Continuity of care on discharge

Patients prioritised a service that shared all information on treatment and preferences with their GP (*β* 0.29, 95% CI 0.05–0.52) and were willing to travel 7.25 miles or more to receive this service. 94% of patients indicated a desired outcome of the admission was for their GP to be fully informed about their care (Supplementary file 5). Cognitive interviews revealed this was preferred despite, at times, the nominal nature of a named GP (Table 2, DCE.04) and an assumption that GPs are fully informed as standard practice.

### Stakeholder consultations

Forty-eight of 82 invited practitioners participated in four consultations (median length 2 h, 12 min), and one individual interview. Participants represented disciplines from across the multidisciplinary teams and grades from unregistered health care assistant (*n* = 6) to registered nurses and allied health professionals (AHPs) (*n* = 28). Main disciplines were registered nurses (36.7%) and AHPs (occupational therapists and physiotherapists) (20.4%). Participants were mainly located in the community hospital and/or were organisation wide (*n* = 36). External staff (*n* = 10) included social care practitioners (*n* = 7), mental health nurse (*n* = 1) and service commissioner (*n* = 1) (Supplementary file 6). Practitioners declined primarily citing work commitments or annual leave (*n* = 16).

### Recommendations

The participants generated 602 items and 200 top recommendations across the four groups. Top recommendations concerned key characteristics for evidence-based tools to enhance care processes on addressing clinical uncertainty in the management of frailty (Question 1, 103 recommendations), and requirements to use evidence-based tools in clinical care (Question 2, 97 recommendations).

A final set of 20 recommendations were formed using top recommendations and the practitioners’ narrative within groups (Table 3). Characteristics of tools for clinical practice (Q1) included 13 recommendations (R). Prominent recommendations included enhancing comprehensive assessment by considering multiple health domains and contextual information, such as home environment (R1), and person-centred care by exploring and recording patient preferences and priorities for care (R5). It was also important to practitioners that the tool was simple and concise (R4) and could be interpreted by all staff (R2) to facilitate communication.

**Table 3:** Recommendations generated from stakeholder consultations

**Question 1: What are the key characteristics of tools for clinical practice to enhance care processes of comprehensive assessment, communication with families and continuity of care between hospital and home (or care home)?**		
**Recommendations:**	**Group top priority (n)**	**Participant top priority (n)**
**R1:** The tool needs to be comprehensive by considering social, physical and psychosocial health domains and record contextual information on the patient journey, such as admitted from, home environment, such as lives alone, and who’s important to the patient.	1	16
**R2:** The tool can be understood and interpreted by all staff, patients and family to support understanding on different roles on using the tool in clinical care.	0	16
**R3:** The tool is relevant for the population and context that used with. The tool has demonstrated value to improve care processes and outcomes.	2	11
**R4:** The tool is simple, clear and concise to complete and interpret.	2	11
**R5:** The tool is person-centred. The tool records goals and preferences and priorities for care as discussed with the patient and/or family, such as in the ‘Welcome meeting’ on admission with the patient and/or family	2	9
**R6:** The tool Is used across care settings and travels with the patient at points of transition in care. This provides a common language between care settings and services to share information succinctly about clinical condition, such as frailty level, symptoms and concerns, and patient priorities	0	5
**R7:** The tool is standardised for use nationally. This will support transitions between settings by providing a common language and enable national benchmarking of services to evaluate care processes and outcomes.	2	4
**R8:** The tool is evidence-based, valid and reliable.	1	4
**R9:** The tool fits in routine care and minimises duplicating existing care processes.	1	4
**R10:** The tool is adaptable, able to tailor to the person, context and setting.	1	4
**R11:** The tool can be repeated overtime to monitor and review change in clinical presentation, such as frailty level at baseline on admission and at discharge.	0	4
**R12:** The tool is sensitive to change in the patient’s symptoms and concerns and can be used across conditions.	0	3
**R13:** The tool supports communication with the family about care, treatment, and anticipated outcomes, and fosters engagement in care processes, such as discharge planning.	1	2
**Question 2: How can we enable staff to use tools in clinical practice to enhance care processes of comprehensive assessment, communication with families and continuity of care between hospital and home (or care home)?**		
**Recommendations:**	**Group top priority (n)**	**Participant top priority (n)**
**R14:** Delivering training and ongoing support to use the tool in clinical care using multiple methods. Methods include in-service training with peer support, champions and leaders, and eLearning with supportive materials such as templates and case studies. Training is tailored for the respective level of responsibility, such as health care assistants and registered staff.	3	30
**R15:** Staff have ownership of the tools used in clinical care. Staff understand the meaning, relevance and clinical importance of the tool, how use enhances clinical care and benefits patient. Staff understand their respective role and feel empowered using the tool to improve clinical care and patient outcomes.	2	20
**R16:** The tool is simple, clear and concise to use and interpret in clinical care. This makes it easy to use and easy to insert in assessments tailored to the individual. It is clear how the tools is completed, by which discipline(s), which components and when. The tool is completed in the electronic patient record to support communication within the clinical team, and across services with integrated record systems, such as to view health records.	2	16
**R17:** Time and physical space is provided for staff to complete tools with the patient and/or family, record on the electronic patient record and review with the multi-disciplinary team.	1	4
**R18:** Staff can pilot using the tool and feedback on what works, and changes needed before implementation in clinical care.	0	3
**R19:** Service evaluation on the processes and outcomes of using the tool, such as audit on completion. Findings are fed back to staff to build confidence, see the value of the tool for patient care and sustain use.	0	3
**R20:** The tool respects staff experience, knowledge and skills.	1	1

Six recommendations detailed requirements for use of tools in clinical care (Q2). Implementing tools required a multiple component training programme comprising in-service training, such as peer support and champions, and formal training, like eLearning (R14). Staff required a sense of ownership of tools to tailor use to the local context (R15). Using nationally standardised tools increased the value of tools to improve care processes and outcomes, and implementation in clinical care (R7). To maximise the impact of using tools on care processes implementation of tools across care settings was required to create a succinct common language between services and enable communication and continuity of care at points of transition in care setting (R6).

The focus group findings illuminated understanding on the recommendations and using evidence-based tools in community hospitals. Key themes comprised: ‘Managing an increasingly older population and uncertain outcomes’; ‘Continuous communication to manage care’; and ‘Using evidence-based tools to deliver continuity of care’. The themes were explored within the theoretical domains of uncertainty of ‘*comprehensive assessment’, ‘communication’* and *‘continuity of care’.*

### Comprehensive assessment: ‘Managing an increasingly older population and uncertain outcomes’

#### Change in patient care needs and practice with an increasingly older population

Key to managing patients multiple and changeable care needs was comprehensive assessment and multi-disciplinary review. Use of standardised tools was a way to support assessment to encompass the multiple health domains, and communication within the team on goals of care. Patients admitted to a community hospital were described as ‘medically complex’ with multiple conditions and often on a ‘knife edge’ with minor events triggering decline and requirement for medical intervention to stabilise and prevent transfer to the acute hospital (Table 2, SC.01). The often rapid transition from acute hospital to the community hospital increased the medical complexity, management and uncertainty of the illness trajectory and outcomes of care. The patient population were mainly older aged 80 years and over with increasing risk for end of life living with co-morbidities and frailty with multiple care needs across health domains. Cognitive impairment, often described as undiagnosed dementia by practitioners, was common and required close involvement of the family to communicate patient’s wishes and support decision making.

#### Person-centred focus on gathering key information

Practitioners discussed the challenges in clinical care to meet the needs of an increasingly older population and address clinical uncertainty in managing frailty and multiple conditions. Managing care was more than using a ‘simple tool’, with requirement for inter-disciplinary working to agree plan of care and review goals and not a ‘tick-box kind of thing’ (Table 2, SC.02). However, use of standardised templates, such as in a ‘Welcome meeting’ with the patient and/or family could prompt identification of patient’s preferences and priorities asking ‘what matters to you’, and understand and manage expectations, plan goals of care and discharge (Table 2, SC.03). Importantly, this enabled family members to advocate for the patient, particularly if cognitive impairment limited ability to communicate wishes. These processes supported practitioners to identify and foster relationships with individuals important in the patient’s care.

### Communication: ‘Continuous communication to manage care’

#### Continuous communication with the family to support and inform care planning

Use of evidence-based tools to support communication with the family required tools that could be easily understood by all, including ‘*a bank member of staff on a Sunday’* or family member. This reduced reliance on one key member of staff to communicate with the family. Evidence-based tools needed to enhance continuous communication with the family to facilitate shared understanding of care plans, including escalation plans to manage decline, the intended outcomes of care and discharge planning. Communication with the family was a vital component to managing uncertainty by facilitating a shared understanding of treatment plans and outcomes. These were often ‘*challenging conversations’* to have (Table 2, SC.04). Standardised processes of a ‘Welcome meeting’ and assessments established contact with the family and identified key members to communicate with.

#### Supporting patient’s psychological adaption to change in ‘what I can do’

Managing patients’ care was complex requiring careful communication to support patients’ psychological adaption to change in level of function and expectations of likely level of recovery. To support the patient, practitioners worked with them, and the family, to build understanding about uncertain illness trajectories and changing care needs. Communication required honest conversations around anticipated level of recovery (Table 2, SC.05) and supportively educating patients that there is not ‘*a magic front door at home that cures all ills’* at home (Table 2, SC.06)*.*

### Continuity of care: ‘Using evidence-based tools to enhance continuity of care’

#### Managing care at points of transition in care settings

Tools used to support continuity of care at points of transition needed to be shared and understood across care settings, by ‘*be* [ing] *moveable between care settings’* or able to travel with the person, such as a ‘*health passport … detailing vital information’* (Table 2, SC.07 and SC.08). This required tools to be implemented across care settings for the information to be *‘fluent and the same’* to communicate preferences for care and treatment and maintain continuity of care (Table 2, SC.08). Tools between care settings needed to record information clearly and succinctly to be understood by ‘*the paramedic at 2am’*. Standardised, relevant information clearly recorded was a way to reduce the family and/or patient requirement to relay information about the patient’s conditions and care.

#### Tension between nationally standardised tools and flexibility in use

Nationally standardised tools were advocated as a way to communicate between services and clinical teams by creating a shared, standardised language understood by all. Implementation of evidence-based tools at a national and organisational level augmented confidence in the value of using tools to enhance care processes and outcomes, with tools being perceived as ‘*fit for purpose’* (Table 2, SC.09), in turn increasing staff commitment to use in clinical care. Using national tools was considered advantageous to support evaluation of care both at the individual patient level and service level by contributing to national audits and benchmarking on care processes and outcomes. Concomitantly, how tools were used required flexibility to allow for clinical judgement and tailoring to the individual patient and clinical context. This required a balance between flexibility and standardisation to ‘*avoid a postcode lottery’* and to ensure completed ‘*accurately’* (Table 2, SC.09 and SC. 10).

## Discussion

**Fig. 3:**
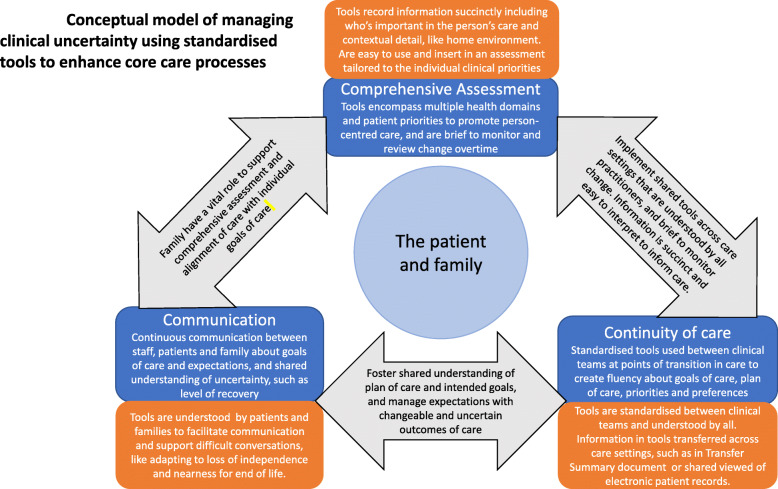
Conceptual model of the management of clinical uncertainty using standardised tools to enhance core care processes

We identified patients’ and practitioners’ preferences and priorities to manage clinical uncertainty using evidence-based tools to develop our conceptual model (Fig. 3). Findings from this study extend Nicholson et al.’s [35] model of supportive care for older people with frailty in acute hospital developed from a systematic review. Our model places central importance on the patient and family and adds applied supportive mechanisms to pursue patient preferences and priorities for care processes within each domain of clinical uncertainty. The model was developed through the convergence of uniform priorities for patients and practitioners; quantitative findings resonated with qualitative data. The DCE and stakeholder consultations exposed the vital role family play to ensure continuity of care at points of transition between care settings for patients with clinically uncertain illness trajectories. Patients prioritised enhanced care processes that involved their family in their care. They specifically preferred processes that maximised involvement of the family in ways that minimised demands on them, such as reduced distance to travel and communication at key points on the care trajectory. The priority for practitioners was for the family to inform understanding of the patient’s care needs and preferences and to support discharge planning. The family had a vital role supporting the patient to adapt psychologically to change. Practitioners identified the potential for evidence-based tools to enhance communication with the family and continuity of care for patients transitioning between care settings. Standardising tools across clinical teams could support the communication of complex information and reduce duplication of work by creating a fluency in understanding and interpretation.

Continuous communication between practitioners and patients and family members is central to manage expectations of care and foster a shared understanding of uncertainty. Our findings highlighted a divergence between the patients’ preferences and practitioners’ knowledge and evidence. Patients desired, and often expected, to regain full function. However, practitioners spoke of uncertainty as to level of functional recovery, and the importance of supporting patients and families to adjust expectations and adapt to change in function. Practitioners indicated a need for tools to support these challenging conversations. However, our systematic review [12] identified limited evidence on tools to support communication with patients and families in addressing clinical uncertainty. These types of relational-conversations are important for older adults following hospital admission to understand changes in functional level and likely recovery of ‘getting back to normal’ or adapting to ‘finding a new normal’ [36]. Poor understanding and management of expectations leads to worse psychological outcomes for patients and families, such as anxiety and distress [4, 37]. Having a shared understanding of the uncertainty of the patient’s condition could enable the family to adjust expectations of level of recovery and support the patient’s adaptation to change in function as well as continuity of care. For older people, psychological adjustment to conditions may be a lower priority than problems that hinder their independence [38]. Understanding the patient’s priorities for care is key to understand how older patients prioritise their multiple health conditions. Yet, only 56% of patients reported being asked ‘what matters to you’ to inform comprehensive assessment, the focus of care and the conversations pursued. ‘Ongoing conversations’ with the patient and the family to ensure alignment of care to patient preferences and ensure family are ‘kept in the loop’. This continuous communication could support the family to ‘hold’ the uncertainty of the patient’s outcomes of care, rather than seeking to resolve it, building on research with care home staff [11].

Family were considered to have a key role advocating for patients, acting as the ‘glue’ to keep care aligned to patient preferences. A finding that is echoed in interviews with older people about their family carers [39], and reaffirming that the patient and family need to viewed as a single unit of care by practitioners delivering palliative care [40]. However, how the family is involved must maximise engagement in ways that is cognisant of minimising demands. Patients in this study prioritised hospitals closer to home to enable family members to visit and preferred less structured communication of staff telephoning the family at key points, such as discharge planning, rather than structured family meetings requiring time to attend with less flexibility. For older adults, family members are often a spouse who may be equally frail, and/or adult children with competing responsibilities and may live further afield. An international survey of family carers of older adults in the last months of life, reveals the high proportion of time spent delivering informal care, such as 7–10 h per week for appointments and 15–18 h for medical procedures [41]. Using a validated carer needs assessment tool in a structured hospital discharge intervention for older adults demonstrated that targeted support improved carer outcomes, such as preparedness for care after hospital discharge [42]. However, if patients deem their family’s involvement to be too demanding they may seek to protect them [43] and alter their care preferences accordingly [40].

The use of evidence-based tools was a priority for practitioners to create a ‘fluency’ within and between clinical teams and with family members adding value to clinical care processes and outcomes of care. For example, the Clinical Frailty Scale (CFS) as a judgement-based frailty tool to identify frailty level 1 (very fit) to 9 (terminally ill) and is used widely in multiple settings with its outcome largely understood by most healthcare practitioners [44]. This creates a standardised method of communicating succinctly a patient’s level of frailty to inform clinical decisions and continuity of care across settings and professional grades. Using a common evidence-based tool across care settings can support continuity in care at points of transition. Information travels with the patient digitally, such as electronic integrated record systems [45], and/or physically, in discharge letters, such as recording on the Transfer Summary the CFS score at admission, discharge and target score to communicate goals and plan of care, or transfer form to communicate preferences for care at the end of life [46]. Working in this way was considered to promote information sharing between services, reduce duplicative work and offer opportunity for reassessment and monitoring across care teams. A nationwide adoption of a specific tool or set of tools to assess defined aspects of care, can provide a universal language for all clinical teams within and across care settings, for example the Australian Palliative Outcomes Collaboration [47]. However, these evidence-based tools also need to be adaptable to the patient and clinical context to support adoption [48]. Importantly, adaptability needs to be achieved without loss to the overall assessment to not comprise the validity of the tool and avoid geographical variations. For example, allowing a ‘cannot assess’ response in the Integrated Palliative care Outcome Scale for Dementia (IPOS-Dem) [49] for symptoms such as nausea when the patient cannot communicate and the practitioner cannot observe.

### Strengths and limitations

A major strength of this study is the integration of the DCE findings on patient priorities to enhance care processes and stakeholder recommendations on using standardised tools to realise these priorities. The study demonstrated feasibility of the DCE both to recruit older patients and to identify patients’ preferences and priorities for enhanced care processes. Intrinsic issues with comprehension due to the hypothetical nature of the DCE were reduced with the skilled support of the research nurse. Our method of data analysis allowed us to explore findings within each method and participant group and triangulate across participant groups. This strengthened confidence in our findings. However, this study has limitations. Our method of recruiting family carers as a dyad with the patient was not feasible, with no family members recruited to the DCE. Recruitment required wider eligibility to include family carers of all patients in the care setting and enable inclusion of perspectives by proxy for patients with impaired capacity, such as with dementia. The absence of family carers limited understanding of their priorities for enhanced care processes, and degree of resonance with the patient perspective, such as, maximising family engagement in ways that minimise demands. The stakeholder consultation included external services, but with limited representation from primary care, such as GPs. Further research is indicated to explore the conceptual model and continuity of care across care settings, such as primary and community health and social care. Finally, we recognise the ethnic homogeneity of the patient participants and hospital locations limits generalisability of findings.

## Conclusions

Our conceptual model supports understanding of patients’ and practitioners’ priorities to manage clinical uncertainty for older people. Families play a vital role in the management of care for patients with clinical uncertainty during transition between hospital and home. Both patients and practitioners prioritised family involvement. Patients preferred any form of enhanced service but prioritised the involvement of their family, especially if minimised demands, such as travel time. Practitioners sought to foster early relationships with families to support communication and decisions about care throughout the patient’s admission to discharge. Our findings and wider evidence suggest that practitioners should work with the family to ‘hold’ the patient’s clinical uncertainty, creating a shared understanding of preferences for care through continuous communication and supporting continuity of care. Use of evidence-based tools could enhance these care processes, when understood by all to communicate complex information succinctly. Using tools that are standardised and shared across care settings could enable communication about preferences, priorities and goals of care to enhance continuity of care and support for the family at points of transition.

## Supplementary Information

**Additional file 1.**

## Data Availability

The datasets used and analysed during the current study are available from the corresponding author on reasonable request.

## Abbreviations

AHP: Allied health professional
DCE: Discrete choice experiment
GP: General practitioner
IPOS-Dem: Integrated palliative care outcome scale for dementia
PACE: Psychosocial assessment and communication evaluation
UK: United Kingdom

## References

[1] Fried LP, Tangen CM, Walston J, Newman AB, Hirsch C, Gottdiener J, Seeman T, Tracy R, Kop WJ, Burke G, McBurnie MA (2001). Frailty in older adults: evidence for a phenotype. J Gerontol A Biol Sci Med Sci.

[2] Gill TM, Gahbauer EA, Han L, Allore HG (2010). Trajectories of disability in the last year of life. N Engl J Med.

[3] Clegg A, Young J, Iliffe S, Rikkert MO, Rockwood K (2013). Frailty in elderly people. Lancet..

[4] Thorne SE, Bultz BD, Baile WF (2005). Is there a cost to poor communication in cancer care?: a critical review of the literature. Psychooncology..

[5] Johnson Wright L, Afari N, Zautra A (2009). The illness uncertainty concept: a review. Curr Pain Headache Rep.

[6] Etkind SN, Karno J, Edmonds PM, Carey I, Murtagh FEM (2015). Supporting patients with uncertain recovery: the use of the AMBER care bundle in an acute hospital. BMJ Support Palliat Care.

[7] Mishel MH (1981). The measurement of uncertainty in illness. Nurs Res.

[8] Mishel MH (1988). Uncertainty in illness. J Nurs Scholarsh.

[9] Mishel MH (1990). Reconceptualization of the uncertainty in illness theory. Image J Nurs Sch.

[10] Etkind SN, Bristowe K, Bailey K, Selman LE, Murtagh FE (2017). How does uncertainty shape patient experience in advanced illness? A secondary analysis of qualitative data. Palliat Med.

[11] Goodman C, Froggatt K, Amador S, Mathie E, Mayrhofer A (2015). End of life care interventions for people with dementia in care homes: addressing uncertainty within a framework for service delivery and evaluation. BMC Palliat care.

[12] Ellis-Smith C, Tunnard I, Dawkins M, Wei G, Higginson IJ, Evans CJ. Managing clinical uncertainty in older people towards the end of life: a systematic review of tools. Under review at BMC Palliative Care. https://kclpure.kcl.ac.uk/portal/en/publications/managing-clinical-uncertainty-in-older-people-towards-the-end-of-life-asystematic-review-of-personcentred-tools(a9e4a34b-6551-46de-a0dc-9a8426691c1b).html. 10.1186/s12904-021-00845-9 PMC8532380 34674695

[13] Ellis G, Whitehead MA, O'Neill D, Langhorne P, Robinson D. Comprehensive geriatric assessment for older adults admitted to hospital. Cochrane Database Syst Rev. 2011;(7):CD006211. 10.1002/14651858.CD006211.pub2 PMC4164377 21735403

[14] Dudgeon D (2018). The impact of measuring patient-reported outcome measures on quality of and access to palliative care. J Palliat Med.

[15] Etkind SN, Daveson BA, Kwok W, Witt J, Bausewein C, Higginson IJ, Murtagh FEM (2015). Capture, transfer, and feedback of patient-centered outcomes data in palliative care populations: does it make a difference? A systematic review. J Pain Symptom Manag.

[16] Wendrich-van Dael A, Bunn F, Lynch J, Pivodic L, Van den Block L, Goodman C (2020). Advance care planning for people living with dementia: an umbrella review of effectiveness and experiences. Int J Nurs Stud.

[17] Koffman J, Yorganci E, Yi D, Gao W, Murtagh F, Pickles A, Barclay S, Johnson H, Wilson R, Sampson L, Droney J, Farquhar M, Prevost T, Evans CJ (2019). Managing uncertain recovery for patients nearing the end of life in hospital: a mixed-methods feasibility cluster randomised controlled trial of the AMBER care bundle. Trials..

[18] Higginson IJ, Koffman J, Hopkins P, Prentice W, Burman R, Leonard S, Rumble C, Noble J, Dampier O, Bernal W, Hall S, Morgan M, Shipman C (2013). Development and evaluation of the feasibility and effects on staff, patients, and families of a new tool, the psychosocial assessment and communication evaluation (PACE), to improve communication and palliative care in intensive care and during clinical uncertainty. BMC Med.

[19] Payne S, Kerr C, Hawker S, Seamark D, Davis C, Roberts H, Jarrett N, Roderick P, Smith H (2004). Community hospitals: an under-recognized resource for palliative care. J R Soc Med.

[20] Evans CJ, Potts L, Dalrymple U, Pring A, Verne J, Higginson IJ (2021). Characteristics and mortality rates among patients requiring intermediate care: a national cohort study using linked databases. BMC Med.

[21] Sezgin D, O’Caoimh R, Liew A, O’Donovan MR, Illario M, Salem MA, Kennelly S, Carriazo AM, Lopez-Samaniego L, Carda CA, Rodriguez-Acuña R, Inzitari M, Hammar T, Hendry A, all EU ADVANTAGE Joint Action Work Package 7 partners (2020). The effectiveness of intermediate care including transitional care interventions on function, healthcare utilisation and costs: a scoping review. Eur Geriatr Med.

[22] Evans CJ, Ison L, Ellis-Smith C, Nicholson C, Costa A, Oluyase AO (2019). Service delivery models to maximize quality of life for older people at the end of life: a rapid review. Milbank Q.

[23] Winpenny EM, Corbett J, Miani C, King S, Pitchforth E, Ling T, van Teijlingen E, Nolte E (2016). Community hospitals in selected high income countries: a scoping review of approaches and models. Int J Integr Care.

[24] Pitchforth E, Nolte E, Corbett J, Miani C, Winpenny E, van Teijlingen E (2017). Health services and delivery research. Community hospitals and their services in the NHS: identifying transferable learning from international developments – scoping review, systematic review, country reports and case studies.

[25] Ev E, Altman DG, Egger M, Pocock SJ, Gøtzsche PC, Vandenbroucke JP (2007). Strengthening the reporting of observational studies in epidemiology (STROBE) statement: guidelines for reporting observational studies. BMJ..

[26] Walkley R. West Sussex Joint Strategic Needs Assessment Briefing: Indices of deprivation 2019. In: Public Health and Social Research Unit WSCC, editor. https://jsna.westsussex.gov.uk/assets/core/Briefing-West-Sussex-IMD-2019.pdf2019.

[27] Website WSJ. Population Estimates West Sussex JSNA Website 2020 [Available from: https://jsna.westsussex.gov.uk/core/population-data/estimates/.

[28] Lancaster KJ (1966). A new approach to consumer theory. J Polit Econ.

[29] Abernethy AP, Shelby-James T, Fazekas BS, Woods D, Currow DC (2005). The Australia-modified Karnofsky performance status (AKPS) scale: a revised scale for contemporary palliative care clinical practice [ISRCTN81117481]. BMC Palliative care..

[30] Goodman CaE CJ. Focus Groups. In: Gerrish K, Lathlean J, Cormack D, editors. The Research Process in Nursing. 7th ed. Chichester: Wiley; 2015.

[31] Evans CJ, Benalia H, Preston NJ, Grande G, Gysels M, Short V, Daveson BA, Bausewein C, Todd C, Higginson IJ, MORECare (2013). The selection and use of outcome measures in palliative and end-of-life care research: the MORECare international consensus workshop. J Pain Symptom Manag.

[32] Braun V, Clarke V (2006). Using thematic analysis in psychology. Qual Res Psychol.

[33] O'Cathain A, Murphy E, Nicholl J (2010). Three techniques for integrating data in mixed methods studies. Bmj..

[34] Ritchie J, Spencer L, ABaR B (1994). Qualitative data analysis for applied policy research. Analyzing qualitative data.

[35] Nicholson C, Morrow EM, Hicks A, Fitzpatrick J (2017). Supportive care for older people with frailty in hospital: an integrative review. Int J Nurs Stud.

[36] Etkind SN, Lovell N, Nicholson CJ, Higginson IJ, Murtagh FE (2019). Finding a 'new normal' following acute illness: A qualitative study of influences on frail older people's care preferences. Palliat Med.

[37] Wright L, Afari N, Zautra A (2009). The illness uncertainty concept: a review. Curr Sci Inc.

[38] Junius-Walker U, Schleef T, Vogelsang U, Dierks M-L (2019). How older patients prioritise their multiple health problems: a qualitative study. BMC Geriatr.

[39] Andersen HE, Hoeck B, Nielsen DS, Ryg J, Delmar C. Caring responsibility from the perspectives of older persons whose adult children are their caregivers. Int J Older People Nurs. 2020;15:e12335. 10.1111/opn.12335. 10.1111/opn.12335 32716593

[40] Etkind SN, Bone AE, Lovell N, Higginson IJ, Murtagh FEM (2018). Influences on care preferences of older people with advanced illness: a systematic review and thematic synthesis. J Am Geriatr Soc.

[41] Higginson IJ, Yi D, Johnston BM, Ryan K, McQuillan R, Selman L, Pantilat SZ, Daveson BA, Morrison RS, Normand C (2020). Associations between informal care costs, care quality, carer rewards, burden and subsequent grief: the international, access, rights and empowerment mortality follow-back study of the last 3 months of life (IARE I study). BMC Med.

[42] Toye C, Parsons R, Slatyer S, Aoun SM, Moorin R, Osseiran-Moisson R, Hill KD (2016). Outcomes for family carers of a nurse-delivered hospital discharge intervention for older people (the further enabling Care at Home Program): single blind randomised controlled trial. Int J Nurs Stud.

[43] Payne S, Hawker S, Kerr C, Seamark D, Roberts H, Jarrett N, Smith H (2007). Experiences of end-of-life care in community hospitals. Health Soc Care Community.

[44] Church S, Rogers E, Rockwood K, Theou O (2020). A scoping review of the clinical frailty scale. BMC Geriatr.

[45] Briggs AM, Valentijn PP, Thiyagarajan JA, Araujo de Carvalho I (2018). Elements of integrated care approaches for older people: a review of reviews. BMJ Open.

[46] Zafirau WJ, Snyder SS, Hazelett SE, Bansal A, McMahon S (2012). Improving transitions: efficacy of a transfer form to communicate patients' wishes. Am J Med Qual.

[47] Currow DC, Allingham S, Yates P, Johnson C, Clark K, Eagar K (2015). Improving national hospice/palliative care service symptom outcomes systematically through point-of-care data collection, structured feedback and benchmarking. Support Care Cancer.

[48] Boyce MB, Browne JP, Greenhalgh J (2014). The experiences of professionals with using information from patient-reported outcome measures to improve the quality of healthcare: a systematic review of qualitative research. BMJ Qual Saf.

[49] Ellis-Smith C, Evans CJ, Murtagh FEM, Henson LA, Firth AM, Higginson IJ, Daveson BA, BuildCARE (2016). Development of a caregiver-reported measure to support systematic assessment of people with dementia in long-term care: the integrated palliative care outcome scale for dementia. Palliat Med.

