# Supplementary material for: Preferences and priorities to manage clinical uncertainty for older people with frailty and multimorbidity: a discrete choice experiment and stakeholder consultations
Source: BMC Geriatr. 2021 Oct 14;21:553. doi: 10.1186/s12877-021-02480-8 (PMC8515697; doi:10.1186/s12877-021-02480-8)
Supplement: Supplementary file 1 — Additional file 1. [file 12877_2021_2480_MOESM1_ESM.docx]

## Title page

**Manuscript title:** Preferences and priorities to manage clinical uncertainty for older people with frailty and multimorbidity: A Discrete Choice Experiment and stakeholder consultations

**Authors:**

India Tunnard^1^ india.tunnard@kcl.ac.uk

Deokhee Yi^1^  deok_hee.yi@kcl.ac.uk

Clare Ellis-Smith^1^ alexandra.c.ellis-smith@kcl.ac.uk

Marsha Dawkins^1^ marsha.j.dawkins@kcl.ac.uk

Irene J Higginson^1^  irene.higginson@kcl.ac.uk

Catherine J Evans^1,2^ catherine.evans@kcl.ac.uk

**Affiliations:**

^1^King’s College London, Cicely Saunders Institute of Palliative Care, Policy and Rehabilitation, Bessemer Road, London, SE5 9PJ

^2^ Sussex Community NHS Foundation Trust, Brighton General Hospital, Elm Grove, Brighton, BN2 3EW

### **Corresponding authors:** Catherine Evans [Catherine.evans@kcl.ac.uk](mailto:Catherine.evans@kcl.ac.uk) and India Tunnard [india.tunnard@kcl.ac.uk](mailto:india.tunnard@kcl.ac.uk)

**Tables and figures**:

Figure 1. Patient and carer recruitment for the Discrete Choice Experiment

Table 1. Patient characteristics for the Discrete Choice Experiment

Figure 2. Preference weights from Discrete Choice Experiment

Table 2. Illustrative quotes from the Discrete Choice Experiment cognitive interviews and stakeholder consultations

Table 3. Recommendations generated from the stakeholder consultations

Figure 3. Conceptual model of the management of clinical uncertainty using standardised tools

**Additional files**

**Additional files to:** Tunnard I, Yi DK, Ellis-Smith C, Dawkins M, Higginson IJ and Evans CJ, ‘Preferences and priorities to manage clinical uncertainty for older people with frailty and multimorbidity: A Discrete Choice Experiment and stakeholder consultations’

Contents

[Part A – Reporting guideline 3](#_Toc78287407)

[Supplementary file 1. STROBE Statement checklist for reports of cross-sectional studies 3](#_Toc78287408)

[Part B - Additional methods 5](#_Toc78287409)

[Supplementary file 2. DCE survey design and administration 5](#_Toc78287410)

[Supplementary file 3. Framework method of data analysis 7](#_Toc78287411)

[Part C - Additional results 8](#_Toc78287412)

[Supplementary file 4. Characteristics of DCE patient participants and non-participants 8](#_Toc78287413)

[Supplementary file 5. DCE descriptive data on formal care pre-admission, discussions about care during admission, and patient priorities from the admission 10](#_Toc78287414)

[Supplementary file 6. Stakeholder consultation participants by hospital site 12](#_Toc78287415)

## Tables and figures

[Table S1: DCE attributes and levels 5](#_Toc78287452)

[Table S2: Framework data analysis stages and application 7](#_Toc78287453)

[Table S3: Characteristics DCE participants and non-participants 8](#_Toc78287454)

[Table S4: Formal care provided pre-admission at home (or care home) 10](#_Toc78287455)

[Table S5: Care and treatment during admission 10](#_Toc78287456)

[Table S6: Priorities for patients during admission 10](#_Toc78287457)

[Table S7: Patient priority outcomes from admission 11](#_Toc78287458)

[Table S8: Characteristics of stakeholder participants 12](#_Toc78287459)

[Figure S1: Example of a DCE choice set 6](#_Toc78287464)

# Part A – Reporting guideline

## Supplementary file 1. STROBE Statement checklist for reports of cross-sectional studies

|  | Item No | Recommendation | Page No |
| --- | --- | --- | --- |
| **Title and abstract** | 1 | (*a*) Indicate the study’s design with a commonly used term in the title or the abstract | 1, 3 |
|  |  | (*b*) Provide in the abstract an informative and balanced summary of what was done and what was found | 3-4 |
| Introduction | | | |
| Background/rationale | 2 | Explain the scientific background and rationale for the investigation being reported | 4-6 |
| Objectives | 3 | State specific objectives, including any prespecified hypotheses | 6 |
| Methods | | | |
| Study design | 4 | Present key elements of study design early in the paper | 6-7 |
| Setting | 5 | Describe the setting, locations, and relevant dates, including periods of recruitment, exposure, follow-up, and data collection | 7-9 |
| Participants | 6 | (*a*) Give the eligibility criteria, and the sources and methods of selection of participants | 7-8 |
| Variables | 7 | Clearly define all outcomes, exposures, predictors, potential confounders, and effect modifiers. Give diagnostic criteria, if applicable | 7-9 |
| Data sources/ measurement | 8* | For each variable of interest, give sources of data and details of methods of assessment (measurement). Describe comparability of assessment methods if there is more than one group | 7-9 |
| Bias | 9 | Describe any efforts to address potential sources of bias | n/a |
| Study size | 10 | Explain how the study size was arrived at | 7-9 |
| Quantitative variables | 11 | Explain how quantitative variables were handled in the analyses. If applicable, describe which groupings were chosen and why |  |
| Statistical methods | 12 | (*a*) Describe all statistical methods, including those used to control for confounding | 9-10 |
|  |  | (*b*) Describe any methods used to examine subgroups and interactions | n/a |
|  |  | (*c*) Explain how missing data were addressed | 9 |
|  |  | (*d*) If applicable, describe analytical methods taking account of sampling strategy | n/a |
|  |  | (*e*) Describe any sensitivity analyses | n/a |
| Results | | | |
| Participants | 13* | (a) Report numbers of individuals at each stage of study—eg numbers potentially eligible, examined for eligibility, confirmed eligible, included in the study, completing follow-up, and analysed | 10-12, 15 |
|  |  | (b) Give reasons for non-participation at each stage | 13 |
|  |  | (c) Consider use of a flow diagram | 11 |
| Descriptive data | 14* | (a) Give characteristics of study participants (eg demographic, clinical, social) and information on exposures and potential confounders | 10-12, 15  and additional file 6 |
|  |  | (b) Indicate number of participants with missing data for each variable of interest | 13 |
| Outcome data | 15* | Report numbers of outcome events or summary measures | n/a |
| Main results | 16 | (*a*) Give unadjusted estimates and, if applicable, confounder-adjusted estimates and their precision (eg, 95% confidence interval). Make clear which confounders were adjusted for and why they were included | 11-19 |
|  |  | (*b*) Report category boundaries when continuous variables were categorized | n/a |
|  |  | (*c*) If relevant, consider translating estimates of relative risk into absolute risk for a meaningful time period | n/a |
| Other analyses | 17 | Report other analyses done—eg analyses of subgroups and interactions, and sensitivity analyses | n/a |
| Discussion | | | |
| Key results | 18 | Summarise key results with reference to study objectives | 19-20 |
| Limitations | 19 | Discuss limitations of the study, taking into account sources of potential bias or imprecision. Discuss both direction and magnitude of any potential bias | 23 |
| Interpretation | 20 | Give a cautious overall interpretation of results considering objectives, limitations, multiplicity of analyses, results from similar studies, and other relevant evidence | 19-23 |
| Generalisability | 21 | Discuss the generalisability (external validity) of the study results | 23 |
| Other information | | | |
| Funding | 22 | Give the source of funding and the role of the funders for the present study and, if applicable, for the original study on which the present article is based | 25 |

*Give information separately for exposed and unexposed groups.

**Note:** An Explanation and Elaboration article discusses each checklist item and gives methodological background and published examples of transparent reporting. The STROBE checklist is best used in conjunction with this article (freely available on the Web sites of PLoS Medicine at http://www.plosmedicine.org/, Annals of Internal Medicine at http://www.annals.org/, and Epidemiology at http://www.epidem.com/). Information on the STROBE Initiative is available at www.strobe-statement.org.

# Part B - Additional methods

## Supplementary file 2. DCE survey design and administration

The DCE survey contained five attributes: *Timing of communication with patient*; *Topics to discuss with patient*; *Timing and mode of communication with family*; *Communication with GP*; and *Willingness to travel to the community hospital*. 3-6 Levels were determined for each attribute (see appendix X: DCE attributes and levels). A combination of all attributes and their levels produced 1,152 (3 Χ 4 Χ 6 Χ 4 X 4) profiles and we created 24 choice sets using a fractional factorial design with a D-optimal design strategy, after removing implausible combinations. To further reduce the number of choices presented to each participant, we used block design by randomisation. Respondents faced eight choice sets and one warm-up at the beginning which was not used in the analysis. We piloted and reviewed the initial DCE sets and their relevance and feasibility with experts from the Project Steering Group and Patient and Public Involvement (PPI) members of the Independent Advisory Group (four men and one woman, aged over 70 years). They completed the DCE and reviewed the number of choice sets, the language and choices offered.

A trained research nurse guided the participants through the DCE by reading aloud a written vignette to familiarize participants with the concept of ‘clinical uncertainty’ in relation to an admission to a community hospital. The DCE was administered using cards presenting the attributes and levels to participants and read aloud to enhance comprehension.

Table S1: DCE attributes and levels

| **Attribute** | **Levels** |
| --- | --- |
| When to discuss concerns and preferences | On admission |
|  | On admission and when planning to go home |
|  | On admission, when unwell and during planning to go home |
| Topics to discuss with staff | Discomfort |
|  | Discomfort and what is important to patient |
|  | Discomfort, what is important to the patient and their worries |
|  | Discomfort, what is important to the patient, and their and their family’s concerns |
| Communication with family (or those close to them) | No communication |
|  | By phone when admitted |
|  | By phone when discharged |
|  | By phone when admitted and before discharged |
|  | Staff available to speak to family when visiting |
|  | Family invited to meet with clinicians |
| Information to be shared with GP | Medical conditions, care and medications |
|  | Medical conditions, care, medications and preference for care in the future |
|  | Medical conditions, care, medications and how to manage care if unwell |
|  | Medical conditions, care, medications, preference for care in the future and how to manage care if unwell |
| Distance to community hospital (miles) | 5 |
|  | 8 |
|  | 15 |
|  | 30 |

Figure S1: Example of a DCE choice set


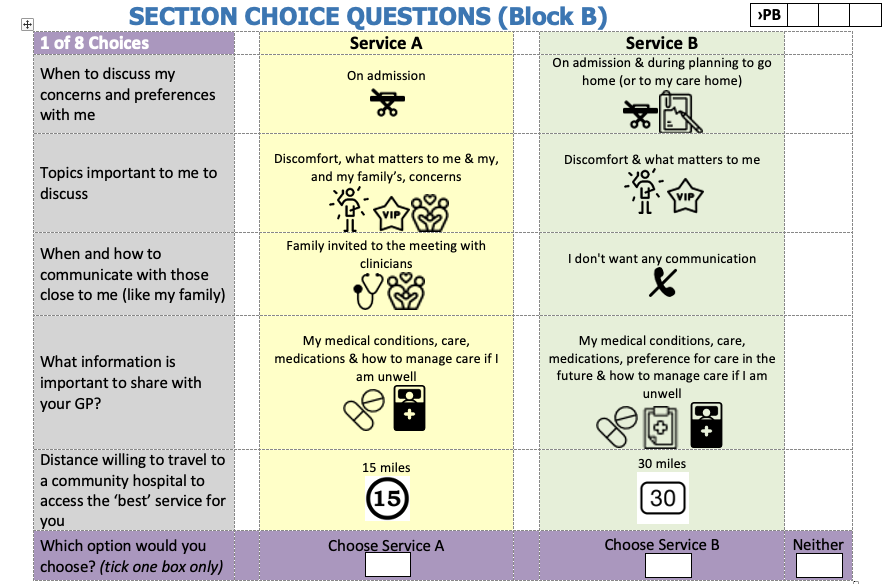


## Supplementary file 3. Framework method of data analysis

Table S2: Framework data analysis stages and application

| **Stages** | **Application** |
| --- | --- |
| Familiarisation | The researcher immersed themselves in the raw data from the focus groups, including transcriptions and recordings. Familiarisation occurred through re-reading of transcripts and noting recurrent themes. |
| Identifying a thematic framework | A thematic framework was developed through a priori questions derived from the aims and objectives of the research, familiarisation with data and discussion amongst research team. The framework was used to identify all key themes and concepts within the data. |
| Indexing | The thematic framework was applied to the transcripts. Descriptions were used to elaborate on short index headings. The NVivo 12 software was used during this stage of analysis. To ensure rigour, a proportion of the indexing (20%) was checked by a second researcher |
| Charting | Data were charted to reflect the area of the thematic framework they related to forming matrices. |
| Mapping and interpretation | Finally, the framework matrices were explored, and study findings interpreted. The researcher was cognisant of areas of convergence and divergence. |

# Part C - Additional results

## Supplementary file 4. Characteristics of DCE patient participants and non-participants

Table S3: Characteristics DCE participants and non-participants

|  | **Participants**  **(n=33)** | **Non-participants**  **(n=52)** |
| --- | --- | --- |
| Mean age (years) | 84 | 84 |
| Gender n (%) |  |  |
| - Female | 25 (75.8) | 35 (67.3) |
| - Male | 8 (24.2) | 17 (32.7) |
| Identified carer n (%) | 4 (12.1) | 12 (23.1) |
| **Admitted from:** | | |
| Home | 1 (3) | 4 (7.7) |
| Acute hospital | 31 (93.9) | 48 (92.3) |
| Community hospital | 1 (3) | - |
|  | **Patients**  **n (%) of reasons recorded in community hospital notes** | **Non-participants**  **n (%) of reasons recorded in community hospital notes** |
| **Reasons for admission** | | |
| Fall with injury | 17 (40.5) | 24 (38.1) |
| Rehab following fall (includes long lie, no injury stated) and fracture | 10 (23.8) | 12 (19) |
| Rehab following planned Total Knee/Hip Replacement | 2 (4.8) | 6 (9.5) |
| Reduced mobility | 4 (9.5) | 4 (6.3) |
| Pain | 3 (7.1) | 3 (4.8) |
| Post-surgery | 1 (2.4) | 2 (3.2) |
| Urinary Tract Infection | 2 (4.8) | - |
| Self-neglect | - | 2 (3.2) |
| Other (including rehab (not specified), leg ulcer management, amputation, delirium, awaiting specialist review) | 3 (7.1) | 10 (15.9) |
| Missing | - | 1 |
| **Total number of reasons for admission reported** | 42 | 63 |
|  | **Participants**  **n (%) diagnoses reported** | **Non-participants**  **n (%) diagnoses reported** |
| **Diagnoses grouped by ICD-10 codes** | | |
| Circulatory system diseases, I | 32 (24.6) | 58 (30.7) |
| Musculoskeletal diseases, M | 30 (23.1) | 23 (12.2) |
| Endocrine, nutritional and metabolic diseases, E | 17 (13.1) | 9 (4.8) |
| Factors influencing health status and contact with health services, Z | 11 (8.5) | 10 (5.3) |
| Disease of the digestive system, K | 8 (6.2) | 13 (6.9) |
| Disorders of the nervous system, G | 6 (4.6) | 8 (4.2) |
| Genitourinary system diseases, N | 6 (4.6) | 5 (2.6) |
| Symptoms, signs and abnormal clinical findings, not elsewhere classified, R | 5 (3.8) | 9 (4.8) |
| Respiratory diseases, J | 3 (2.3) | 15 (7.9) |
| Cancer, C | 3 (2.3) | 8 (4.2) |
| Skin diseases, L | 3 (2.3) | 5 (2.6) |
| Mental and behavioural disorders, F | 2 (1.5) | 9 (4.8) |
| Blood diseases, D | 2 (1.5) | 3 (1.6) |
| Certain infectious and parasitic diseases, A | 2 (1.5) | 1 (0.5) |
| Eye disease, H | - | 5 (2.6) |
| Disease of the ear, H | - | 3 (1.6) |
| Injury, S | - | 1 (0.5) |
| Missing | - | 4 (2.1) |
| **Total number of diagnoses reported** | 130 | 189 |

## Supplementary file 5. DCE descriptive data on formal care pre-admission, discussions about care during admission, and patient priorities from the admission

Table S4: Formal care provided pre-admission at home (or care home)

| Service provider who helped with health conditions at home (or care home) | Yes  N(%) |
| --- | --- |
| GP | 32(100%) |
| District nurse or community nurse | 12(38%) |
| Specialist nurse (e.g. for Parkinson Disease, Heart Failure, Respiratory, Palliative care/Macmillan Nurse) | 4(13%) |
| Medical Consultant at a hospital | 17(53%) |
| Social worker | 1(3%) |
| Occupational therapist or physio therapist | 15(47%) |
| Others | 14(44%) |

Table S5: Care and treatment during admission

| Things discussed with health care providers | Yes  N(%) |
| --- | --- |
| Asked you about ‘what matters to you’ (or family/friend on my behalf) | 18(56%) |
| Review of non-medical treatment (e.g. walking aid) | 27(84%) |
| Review of medical treatment (e.g. medicines like a tablet) | 29(91%) |
| Review of psychological wellbeing (e.g. anxiety, depression) | 12(38%) |
| Physiotherapy or occupational therapy | 32(100%) |
| Support by a social worker | 7(22%) |
| Talked about preferences and priorities for care in the future | 18(56%) |
| Other | 5(16%) |

Table S6: Priorities for patients during admission

| What is important to you | Yes  N(%) |
| --- | --- |
| To feel that staff understand what matters to me | 31(97%) |
| To have sufficient information about my care and treatment so I understand what to expect (or my family/friend on my behalf) | 30(94%) |
| To be involved in decisions about my care and treatment (or my family/friend speak on my behalf) | 30(94%) |
| To talk about my preferences for care in the future if I am unwell or nearing the end of life (or my family speak on my behalf) | 25(78%) |
| To discuss with my family (or friend) about my care and treatment and what to expect | 24(75%) |
| Other | 5(16%) |

Table S7: Patient priority outcomes from admission

| What do you hope to gain from admission to the community hospital | Yes  N(%) |
| --- | --- |
| To understand better about the health conditions I live with - what to expect and how to manage to keep well, and if unwell | 20(63%) |
| To be able to live as independently as possible at home (or in a care home) and to be able to enjoy social activities | 32(100%) |
| To be able to remain at home (or in the care home) with sufficient support | 31(97%) |
| To have less visits to A&E in the future | 21(66%) |
| To have less admissions to hospital in the future | 25(78%) |
| That my GP is informed about ‘what matters to me’, my conditions and how to manage them to keep me well and if I am unwell | 30(94%) |
| To have talked with staff about my priorities and preferences for care now and in the future, like my wishes for care towards the end of life (or with my family/friend on my behalf) | 16(50%) |
| Other | 4(13%) |

Missing data n=1 participant could not complete DCE.

| **Hospital site ID** | **Number of participants [invited/declined]** | **Role of Participants**  **Internal staff or external providing service to the community hospital** | **Number of recommendations** | |
| --- | --- | --- | --- | --- |
|  |  |  | **Q 1** | **Q 2** |
| 01 | n=17  [invited n=19/  declined n=4] | **Internal staff** (Missing n=1)   - Registered nurse n=8 - AHP n=1 - HCA n=2 - Medical doctor n= 1   **External staff**   - Mental health nurse n=1 - Social care n=3 | 85 | 78 |
| 02 | n=16  Individual interview n=1  [invited n=25/  declined n=5] | **Internal staff**   - Registered nurse n=7 - AHP n=2 - HCA n=3 - Medical doctor n= 1   **External staff**   - Mental health n=1 - Social care n=2 - Commissioner n=1 | 155 | 113 |
| 03 | n=5  [invited n=14  declined n=5] | **Internal staff**   - AHP n=3 - Medical doctor n= 1   **External staff**   - Social care n=1 | 42 | 36 |
| 04 | n=10  [invited n=24/  declined n=2] | **Internal staff** (Missing n=1)   - Registered nurse n=3 - AHP n=4 - HCA n=1   **External staff**   - Social care n=1 | 62 | 31 |
| **Totals** | Participated n=48  [invited n = 82/ declined n= 16 | Internal staff n= 36  External staff n=10  Missing n=2 | 344 | 258 |

## Supplementary file 6. Stakeholder consultation participants by hospital site

Table S8: Characteristics of stakeholder participants

Registered nurses included staff nurse to Advanced Nurse Practitioner; AHP – allied health professional including occupational therapist and physiotherapist; HCA – Health care assistant; Medical doctor including Medical consultant Geriatrician, Ward based General Practitioner and Specialist Medical Doctor; and Social care including registered social worker, social work assistant and carer support practitioner
